# Monitoring Human Babesiosis Emergence through Vector Surveillance New England, USA

**DOI:** 10.3201/eid2002.130644

**Published:** 2014-02

**Authors:** Maria A. Diuk-Wasser, Yuchen Liu, Tanner K. Steeves, Corrine Folsom-O’Keefe, Kenneth R. Dardick, Timothy Lepore, Stephen J. Bent, Sahar Usmani-Brown, Sam R. Telford, Durland Fish, Peter J. Krause

**Affiliations:** Yale School of Public Health, New Haven, Connecticut, USA (M.A. Diuk-Wasser, Y. Liu, T.K. Steeves, C. Folsom-O’Keefe,; S.J. Bent, S. Usmani-Brown, D. Fish, P.J. Krause);; Uppsala University, Uppsala, Sweden (Y. Liu);; Audubon Connecticut, Southbury, Connecticut, USA (C. Folsom-O’Keefe);; Mansfield Family Practice, Storrs, Connecticut, USA (K.R. Dardick);; Nantucket Cottage Hospital, Nantucket, Massachusetts, USA (T. Lepore);; University of Adelaide, Adelaide, South Australia, Australia (S.J. Bent);; Tufts University, Boston, Massachusetts, USA (S.R. Telford III);; L2 Diagnostics, New Haven (S. Usmani-Brown)

**Keywords:** *Ixodes scapularis*, *Borrelia burgdorferi*, *Babesia microti*, tick-borne pathogens, Lyme disease, human babesiosis, parasites, piroplasm, infection prevalence, incidence ratio, emergence, New England, Connecticut, Massachusetts

## Abstract

Human babesiosis is an emerging tick-borne disease caused by the intraerythrocytic protozoan *Babesia microti*. Its geographic distribution is more limited than that of Lyme disease, despite sharing the same tick vector and reservoir hosts. The geographic range of babesiosis is expanding, but knowledge of its range is incomplete and relies exclusively on reports of human cases. We evaluated the utility of tick-based surveillance for monitoring disease expansion by comparing the ratios of the 2 infections in humans and ticks in areas with varying *B. microti* endemicity. We found a close association between human disease and tick infection ratios in long-established babesiosis-endemic areas but a lower than expected incidence of human babesiosis on the basis of tick infection rates in new disease-endemic areas. This finding suggests that babesiosis at emerging sites is underreported. Vector-based surveillance can provide an early warning system for the emergence of human babesiosis.

## Abstract 2

Such surveillance can provide an early warning for emergence of this disease and measure disease underreporting.

Human babesiosis is an emerging tick-borne disease in the United States, caused primarily by the intraerythrocytic protozoan *Babesia microti* and by other *Babesia* species (*1**,**2*). *B. microti* also may be transmitted through infected blood and is the most commonly reported transfusion-transmitted pathogen in the United Sates (*3**,**4*). The outcome of *B. microti* infection varies from asymptomatic or moderate disease in previously healthy persons to severe and sometimes fatal disease in persons who are elderly or immunocompromised (*1*). Fatality rates of 6% to 9% have been reported among hospitalized patients and of ≈20% among those who are immunosuppressed or who experience transfusion-transmitted babesiosis (*3*–*7*). Although *B. microti* shares the same tick vector (*Ixodes scapularis* ticks) and primary reservoir host (*Peromyscus leucopus* mice) (*1**,**8*) as the Lyme disease agent (*Borrelia burgdorferi*), the number of Lyme disease cases reported nationally in 2011 was ≈25 times greater than that of babesiosis cases. This disparity probably results from the more limited geographic distribution of *B. microti,* although lower *B. microti* tick infection prevalence in babesiosis-endemic areas or lower tick-to-human transmission rates may also be contributing factors. Babesiosis has expanded in a pattern similar to that of Lyme disease, albeit at a slower pace (*9**,**10*). Both diseases are following the range expansion of *I. scapularis* ticks over the past 30 years (*9**,**11*). If *B. microti* continues its current rate of expansion, it may ultimately have the same distribution as *B. burgdorferi*.

Current knowledge of the geographic range of babesiosis is incomplete and relies exclusively on human case reports. Case reports are poor indicators of risk for babesiosis because of the low index of suspicion on the part of many physicians and the lack of distinctive clinical signs such as the erythema migrans rash of Lyme disease. An alternative approach to determining infection risk is tick-based surveillance, which is likely to be a more sensitive method for identifying areas where babesiosis is emerging and can be used to estimate zoonotic prevalence in established areas. The incidence of tick-borne disease is primarily determined by 2 factors: human–tick contact rates and the proportion of the tick population that is infected (*12**,**13*). Human–tick contact rates are difficult to measure accurately because they are highly spatially heterogeneous and are determined by complex interactions between human and tick populations, depending on particular tick densities and human behaviors associated with human exposure.

We proposed an integrative measure of tick-borne disease risk that combines tick infection prevalence and human incidence data for an established disease (Lyme disease) and an emerging disease (babesiosis). We hypothesized that the ratio of human Lyme disease to babesiosis incidence rates (hereafter termed “human ratio”) is directly proportional to the prevalence ratio of *I. scapularis* ticks infected with *B. burgdorferi* and that of those infected with *B. microti* (hereafter termed “tick ratio”) in babesiosis – and Lyme disease–endemic areas. We based this hypothesis on the fact that the ratio of incidence rates for >1 infection transmitted by the same tick should depend only on the ratio of tick infection prevalence with the respective pathogens because human–tick contact rates are the same for both. We further hypothesized that the human ratio would be relatively higher than the tick ratio in areas where babesiosis is newly emerging because of underreporting of emerging disease. Accordingly, we determined the relationship between the human ratio and the tick ratio in regions in southern New England where the disease is endemic and emerging.

## Methods

### Study Sites

**Figure 1:**
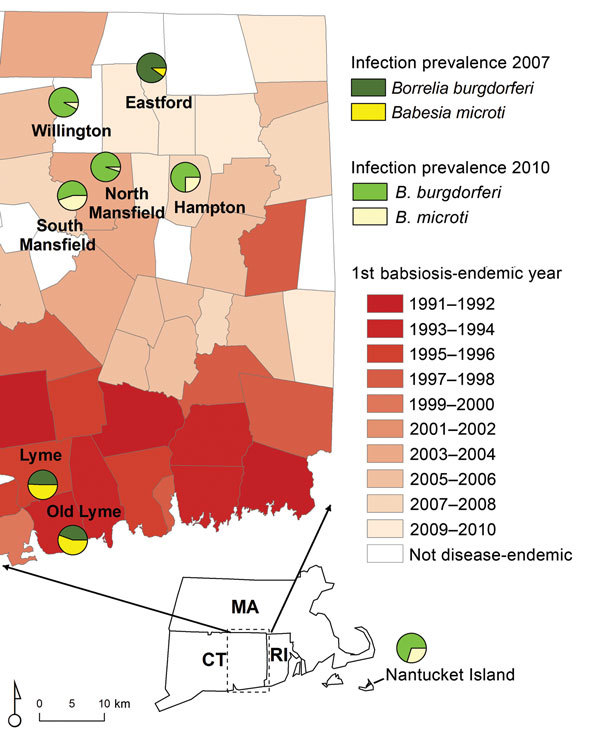
*Borrelia burgdorferi* and *Babesia microti* infection prevalence among humans and *Ixodes scapularis* ticks, eastern Connecticut (CT) and Nantucket, Massachusetts (MA). Shading indicates human babesiosis incidence in study towns by year in which the disease became endemic in the town (defined as the first year babesiosis cases were reported for 2 consecutive years). *I. scapularis* nymphal infection prevalence is shown for *B. microti* and *B. burgdorferi* in Lyme/Old Lyme in 2007 and for Nantucket and northeastern CT in 2010, represented as a pie chart for each sampled location. RI, Rhode Island.

We established study sites in towns in Lyme disease–endemic regions in Connecticut and Massachusetts on the basis of when human babesiosis became endemic. A town was defined as babesiosis-endemic the first time the state public health department reported human babesiosis cases for 2 consecutive years. We identified 2 sites in southeastern Connecticut (Lyme and Old Lyme) and 1 site in Massachusetts (Nantucket) where babesiosis has been endemic for at least a decade. Nantucket was the first area where human babesiosis was identified as endemic in the United States (*14*), and Lyme disease and babesiosis have been highly endemic there for 4 decades. Lyme disease and babesiosis have been endemic in Lyme and Old Lyme for 2 decades (Figure 1, Table 1). We also established 5 study sites in northeastern Connecticut (Eastford, Hampton, North Mansfield, South Mansfield, and Willington), where babesiosis has become endemic within the past 10 years or where it is not yet considered endemic (Figure 1, Table 1).

**Table 1:** Summary of nymphal *Ixodes scapularis* infection with *Babesia microti,* *Borrelia burgdorferi* ,and both, Nantucket Island, Massachusetts, and Connecticut, 2007 and 2010

Region	Year became disease endemic	Year tick sample collected	*B. burgdorferi* infection prevalence (no. positive/no. tested)	*B. microti* infection prevalence (no. positive/no. tested)	Tick ratio*	Co-infection prevalence (no. positive/no. tested)
Nantucket, MA	1969	2010	0.21 (18/87)	0.09 (8/87)	2.33	0.01 (1/87)
Southeastern CT						
Lyme	1996	2007	0.13 (23/182)	0.15 (28/182)	0.82	0.04 (8/182)
Old Lyme	1992	2007	0.20 (13/65)	0.20 (13/65)	1.00	0.06 (4/65)
Combined sites			0.15 (36/247)	0.17 (41/247)	0.88	0.05 (12/247)
Northeastern CT						
Hampton	2007	2010	0.29(43/147)	0.10 14/147)	2.90	0.05 (8/147)
South Mansfield	2002	2010	0.11 (12/111)	0.09 (10/111)	1.22	0.01 (1/111)
North Mansfield	2002	2010	0.13 (18/139)	0.01 (1/138)	13.00	0.00 (0/138)
Willington	Not endemic	2010	0.36 (51/142)	0.03 (4/142)	12.00	0.02 (3/142)
Eastford	Not endemic	2007	0.31 (93/298)	0.03 (10/298)	10.33	0.03 (10/298)
Combined sites			0.26 (217/836)	0.05 (39/836)	5.20	0.03 (24/828)

*Ratio of *I. scapularis* ticks infected with *B. burgdorferi* and that of ticks infected with *B. microti.*

### Prevalence *of B. burgdorferi* and *B. microti* Infection in *I. scapularis* Nymphs

Host-seeking *I. scapularis* nymphs were collected at the study sites in 2007 and 2010 (Table 1). Sampling was conducted once or twice at each site during the peak nymphal host-seeking period from late May to late June. We focused on the nymphal stage of *I. scapularis* because it is the only tick stage with a significant role as a vector for *B. burgdorferi* in North America (*15*). Because of their small size, these nymphs often escape detection long enough to transmit *B. burgdorferi* (*16*). To sample *I. scapularis* nymphs, researchers dragged a 1-m^2^ corduroy cloth over leaf litter along a variable number of 100-m transects and conducted supplemental nonquantitative drags to provide additional ticks (*17*). A total of 1,170 nymphal ticks (87 on Nantucket, 247 in southeastern Connecticut, 836 in northeastern Connecticut) were collected in forested areas and peridomestic habitats adjacent to forests (Table 1). The cloth was inspected for ticks at 20-m intervals, and ticks were preserved in vials containing 70% ethanol. The geographic coordinates of all transects relative to the map datum WGS84 were recorded by using a handheld global positioning system receiver (Garmin, Olathe, KS, USA).

All ticks were identified by using a dissecting microscope and taxonomic keys (*18*). DNA was extracted from all *I. scapularis* nymphs with QIAGEN DNeasy blood and tissue kit (QIAGEN Inc., Valencia, CA, USA) by using a modified protocol (*19*). A nested PCR was performed to amplify the 16S-23S rRNA intergenic spacer region of *B. burgdorferi* by using the primers and protocols developed by Liveris et al. (*20*). Amplicons were visualized on 1% agarose gel by using ethidium bromide. All positive samples were sequenced bi-directionally. *B. burgdorferi* amplicons were typed by comparing them with known genotypes by using BLAST (*21*). Ticks were tested for *B. microti* infection by using a reverse transcription PCR that targets a sequence of the *B. microti* 18S rRNA gene (GenBank accession no. AY144696.1) (*22*). Forward and reverse primers and probe sequences were (5′→ 3′) AACAGGCATTCGCCTTGAAT, CCAACTGCTCCTATTAACCATTACTCT, and 6FAM-CTACAGCATGGAATAATGA-MGBNFQ, respectively. The assay was performed by using Applied Biosystems 7500 Real-Time PCR machine (Foster City, CA, USA). The PCR reaction consisted of TaqMan Universal PCR Master Mix (2X) (with AmpErase, Applied Biosystems), 0.9 μM forward and reverse primers, 0.2 μM Probe, and 5 μL DNA template in a total reaction volume of 25 μL. Ticks were considered positive if they displayed amplification at or before a cycle threshold value of 35.

### Incidence Rates for Babesiosis and Lyme Disease in Humans

We calculated annual incidence rates for babesiosis and Lyme disease in humans for the towns and years in which tick assessments were conducted by using reported cases and mid-year population estimates from the Connecticut Department of Public Health (*23*) and the Massachusetts Department of Public Health (*24*). We also calculated annual incidence rates for 2010 by using clinical diagnosis data from a private physician practice on Nantucket Island with ≈10,000 patients and a practice in Mansfield, Connecticut. The Mansfield practice services a large catchment area that includes 4,000 patients from the towns where ticks were collected for this study (Eastford, Hampton, Mansfield, and Willington).

### Data Analyses

Logistic regression analyses were performed to assess differences in prevalence of infection with *B. burgdorferi* and *B. microti* in *I. scapularis* nymphs and to compare infection prevalence of each of these pathogens among study sites. We derived a tick-infection prevalence ratio (or tick ratio) by dividing the prevalence of *B. burgdorferi* infection by the prevalence of *B. microti* infection from samples from each of the 3 study regions (southeastern Connecticut, Nantucket, and northeastern Connecticut). We derived a disease incidence-rate ratio (or human ratio) by dividing Lyme disease incidence rates by babesiosis incidence rates for state-reported cases by town and for each medical practice. For the combined estimates of the southeastern and northeastern Connecticut regions, the state-reported annual incidence rates were calculated by adding all cases and dividing by the respective mid-year population estimates for the towns where tick samples were collected.

We evaluated the association between the human ratio and tick ratio by fitting a linear regression model. The model included state-reported case data from babesiosis-endemic sites and case diagnoses from both medical practices. These datasets were selected with the assumption that Lyme disease and babesiosis would probably be diagnosed and reported in a similar manner at these sites. Both diseases are well known by the study physicians on Nantucket and in Mansfield and at the study sites where Lyme disease and babesiosis have been endemic for ≥2 decades (Lyme and Old Lyme). We then compared the state-reported human ratio in areas where is babesiosis emerging to its expected value, on the basis of the tick ratio. In areas where babesiosis was emerging, if the value of the human ratio was higher than expected from the tick ratio, underreporting of babesiosis (in comparison to Lyme disease) was probable. Statistics were performed by using Stata 12 Statistical Software (StataCorp. LP, College Station, TX, USA).

## Results

### Prevalence of *B. burgdorferi and B. microti* Infection in *I. scapularis* Nymphs

Mean tick infection prevalence for *B. burgdorferi* was 0.23 ± 0.42 and 0.08 ± 0.26 for all sites and years combined. *B. burgdorferi* tick infection prevalence was higher than *B. microti* tick infection prevalence when all sites were combined but varied significantly by region. *B. burgdorferi* infection was 5.2 times more prevalent than *B. microti* infection in northeastern Connecticut, 2.3 times more prevalent than *B. microti* infection on Nantucket, and was not significantly different in prevalence from *B. microti* infection in southeastern Connecticut (Tables 1, 2). The tick ratio varied from 0.88 to 5.20 across regions (Tables 1, 3). Rates of co-infection with both pathogens varied from 0 to 0.06 across regions.

**Table 2:** Logistic regression comparing the prevalence of *Borrelia burgdorferi* infection with *Babesia microti* infection among *Ixodes scapularis* nymphal ticks, New England*

Prevalence	Odds ratio	SE	*z* score	p value (95% CI)
Nantucket, MA	2.57	1.17	2.08	0.038 (1.050–6.290)
Northeastern CT	7.16	1.30	10.82	0.00 (5.01–10.23
Southeastern CT	0.86	0.21	−0.62	0.53 (0.52–1.39)
All sites combined	3.71	4.8	10.02	0.00 (2.86–4.78)

*Results are shown stratified by region and for all sites combined.

**Table 3:** Comparison between the ratio of Lyme disease and babesiosis incidence in humans and the ratio of *Borrelia burgdo*rferi and *Babesia microti* infection in ticks (‘tick ratio’), New England, 2007 and 2010*

Data source, y	Lyme disease†	Babesiosis†	Human ratio‡	*B. burgdorferi§*	*B. microti§*	Tick ratio¶	Human/tick ratio
Private practice							
Nantucket, 2010	7,500	1,250	6.0	0.21	0.09	2.33	2.6
Northeastern CT, 2010	660	70	9.4	0.26	0.05	5.20	1.8
Reported to CT DPH or MA DPH							
Nantucket, 2010	194	56	3.5	0.21	0.09	2.33	1.5
Northeastern CT, 2010	211	7	30.0	0.26	0.05	5.20	5.8
Southeastern CT, 2007	191	85	2.3	0.15	0.17	0.88	2.6

*CT DPH, Connecticut Department of Public Health; MA DPH, Massachusetts Department of Public Health. †Annual incidence rates of Lyme disease and human babesiosis calculated for the towns and years for which tick assessments were conducted by using private practice or state-reported cases and mid-year population estimates from CT DPH (*23*) and MA DPH (*24*). ‡Ratio of human Lyme disease incidence rates to babesiosis incidence rates. §Nymphal *Ixodes scapularis* tick infection prevalence with *Borrelia burgdorferi *and *Babesia microti.* ¶ Ratio of *I. scapularis* ticks infection prevalence with *B. burgdorferi* and infection prevalence with *B. microti*.

### Incidence Rates for Lyme Disease and Babesiosis in Humans

Incidence rates of Lyme disease in humans ranged from 191 to 211 cases per 100,000 population, based on state-reported data; 660, based on data from the medical practice in northeastern Connecticut; and 3,750, based on data from the medical practice on Nantucket. Incidence rates for human babesiosis ranged from 7 to 85 cases per 100,000 population, based on state reported data; 70, based on data from the northeastern Connecticut medical practice, and 1,250, based on data from the Nantucket medical practice. Lyme disease incidence rates were higher than babesiosis incidence rates in all regions, and the human ratio varied from 2.3 in southeastern Connecticut (state reported) to 30.0 in northeastern Connecticut (state reported) (Table 3).

### Association between Human and Tick Ratios

**Figure 2:**
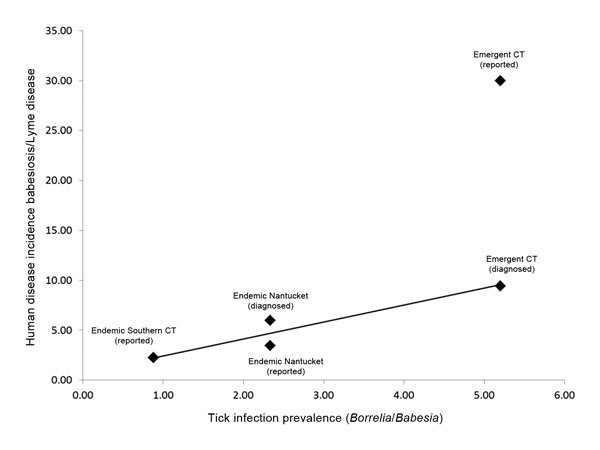
Linear regression model of the human ratio (disease incidence rate ratio) and the tick ratio (tick infection prevalence ratio). The regression model includes state-reported case data from disease-endemic sites and case diagnoses from both medical practices. The human ratio derived from state-reported case data in the emerging area (northeastern Connecticut) is also displayed.

The human ratio was higher than the corresponding tick ratio for both private practice and state reported case data in all regions/datasets (Table 3). A linear regression of the human ratio (combining medical practice and state-reported cases in disease-endemic regions) on the tick ratio yielded the following (Figure 2):

*HR* = 1.95 × *TR* + 0.34 (1)

*HR* = human ratio or human incidence rate ratio

*TR* = tick ratio or tick infection prevalence ratio

Ninety percent of the variance in the human ratio was explained by the tick ratio (*R^2^* = 0.9). The 1.95 regression coefficient indicates that exposure to *B. burgdorferi*–infected ticks was nearly twice as likely to result in a case diagnosis as was exposure to *B. microti*–infected ticks. The reported human ratio from state-reported case data in the babesiosis-emergent area in northeastern Connecticut was higher than expected by the regression model (Figure 2), suggesting underreporting of babesiosis.

## Discussion

We examined the ratio of *B. burgdorferi* infection to *B. microti* infection in humans and ticks and found that in areas where the 2 infections have been endemic for at least 20 years, the human ratio was consistently 2 times higher than the tick ratio. We found a strong positive association between the human and tick ratios, using either state health department data from long-time *B. microti*–endemic states or data from private practices, indicating that the tick ratio can be used to estimate the relative risk for humans of acquiring *B. burgdorferi* and *B. microti* infection. In contrast, the human ratio in *B. microti–*emerging areas in northeastern Connecticut calculated from state-reported data was about 6 times higher than the tick ratio. These larger human ratios in emerging areas probably result from physician underreporting of babesiosis in comparison to Lyme disease cases. The nationally reported Lyme disease and babesiosis case ratio (25:1) is even greater and is probably caused by a combination of underreporting of babesiosis and the wider geographic distribution of *B. burgdorferi*. We conclude that the ratio of ticks infected with *B. burgdorferi* to those infected with *B. microti* may be used as a sensitive surveillance tool to identify new areas where human babesiosis is endemic but has not yet been reported or is underreported.

We found areas in Connecticut where Lyme disease is endemic but babesiosis is absent, or present at very low prevalence. In contrast, no areas where babesiosis is endemic and Lyme disease is not present have been reported. Babesiosis is increasingly being reported from areas where Lyme disease alone was previously endemic. Originally reported from Nantucket Island, Massachusetts, in 1969 (*14*), babesiosis has expanded its geographic range to mainland Connecticut (*9**,**25*), Maine (*26*), Massachusetts (*27**,**28*), New Jersey (*29**,**30*), New York (*7*,*31*), and Rhode Island (*32**,**33*). A similar range expansion is being reported from the Upper Midwest (*34*). As with Lyme disease (*12*,*35*), the emergence of *B. microti* in ticks has resulted in dramatic increases in the number of reported human cases of babesiosis, with a 3-fold increase in Connecticut from 1998 to 2008 (*23*) and a 20-fold increase in the lower Hudson Valley in New York from 2001 to 2008 (*31*). Our finding of a 2.3–3.5 ratio of babesiosis to Lyme disease in babesiosis-endemic areas is consistent with the 1.7 ratio reported in a 10-year prospective study on Block Island, Rhode Island, where babesiosis and Lyme disease have been endemic for >2 decades (*33*) This finding suggests that as *B. microti* expands into areas previously enzootic for *B. burgdorferi* alone, the national 25:1 human ratio is likely to narrow.

The lower incidence of babesiosis in areas where both diseases are endemic is due in part to the lower prevalence of *B. microti* infection in *I. scapularis* nymphs. Less efficient transmission of *B. microti* from infectious hosts to ticks may account for this difference. Mather et al. (*36*) found that 45% of nymphs derived from larvae that fed on *B. microti*–infected *P. leucopus* mice were infected with *B. microti* sporozoites, whereas 92% of nymphs derived from larvae that fed on a *B. burgdorferi*–infected mouse were infected with the spirochete. The decreased ability of *B. microti* to survive in overwintering nymphs compared with that of *B. burgdorferi* appears to further reduce infection prevalence in *I. scapularis* ticks (*37*). Although these laboratory findings are consistent with the higher average prevalence of *B. burgdorferi*, they are not sufficient to explain the large variability observed in prevalence ratios for *B. burgdorferi*/*B. microti* infection in *I. scapularis* nymphs, which ranged from 0.9 to 5.2. Local differences in *I. scapularis* nymphal abundance (*32*,*38*) or the composition of the enzootic host community (*8*) may help explain this variation.

Differences in *B. burgdorferi* and *B. microti* infection prevalence in ticks were ≈2-fold, whereas incidence of human Lyme disease was ≈4-fold higher than that of babesiosis in human babesiosis–endemic areas. Thus, the disparity in Lyme disease/babesiosis human case reporting was about twice that of tick-infection prevalence. This finding may be caused by a combination of decreased ability of nymphs to transmit *B. microti* efficiently to humans (*39*); greater difficulty in diagnosing babesiosis than Lyme disease; and more frequent asymptomatic *Babesia* infection, particularly in the young, immunocompetent population. The rates of asymptomatic infection in adults were 25% for *B. microti* infection and 10% for *B. burgdorferi* infection in previous studies (*33*,*40*). Longitudinal studies of Lyme disease and babesiosis in humans and infection prevalence in ticks are necessary for assessing the relative importance of these factors in determining the ratio of human Lyme disease to babesiosis.

The proposed tick surveillance method can be used to monitor the emergence of babesiosis and other *I. scapularis* tick–borne emerging infections in the eastern United States, such as human granulocytic anaplasmosis (*Anaplasma phagocytophilum*), Powassan virus disease (Powassan virus), and hard tick relapsing fever (*Borrelia miyamotoi*). Vector-based surveillance that compares the incidence of emerging infections to that of Lyme disease can serve as a powerful tool for identifying new disease-endemic areas, leading to early case recognition and reduction in disease risk.

## References

[1] Vannier E, Krause PJ. Human babesiosis. N Engl J Med. 2012;366:2397–407 . 10.1056/NEJMra1202018 22716978

[2] Conrad PA, Kjemtrup AM, Carreno RA, Thomford J, Wainwright K, Eberhard M, Description of *Babesia duncani* n.sp. (Apicomplexa: Babesiidae) from humans and its differentiation from other piroplasms. Int J Parasitol. 2006;36:779–89 . 10.1016/j.ijpara.2006.03.008 16725142

[3] Herwaldt BL, Linden JV, Bosserman E, Young C, Olkowska D, Wilson M. Transfusion-associated babesiosis in the United States: a description of cases. Ann Intern Med. 2011;155:509–19 . 10.7326/0003-4819-155-8-201110180-00362 21893613

[4] US Food and Drug Administration. Biological product and HCT/P deviation reports: annual summary for fiscal year 2008. [cited 2009 Oct]. http://www.fda.gov/BiologicsBloodVaccines/SafetyAvailability/ReportaProblem/BiologicalProductDeviations/ucm169990.htm

[5] Krause PJ, Gewurz BE, Hill D, Marty FM, Vannier E, Foppa IM, Persistent and relapsing babesiosis in immunocompromised patients. Clin Infect Dis. 2008;46:370–6 . 10.1086/525852 18181735

[6] Tonnetti L, Eder AF, Dy B, Kennedy J, Pisciotto P, Benjamin RJ, Transfusion-transmitted *Babesia microti* identified through hemovigilance. Transfusion. 2009;49:2557–63 . 10.1111/j.1537-2995.2009.02317.x 19624607

[7] White DJ, Talarico J, Chang HG, Birkhead GS, Heimberger T, Morse DL. Human babesiosis in New York State—review of 139 hospitalized cases and analysis of prognostic factors. Arch Intern Med. 1998;158:2149–54 . 10.1001/archinte.158.19.2149 9801183

[8] Spielman A, Etkind P, Piesman J, Ruebush TK II, Juranek DD, Jacobs MS. Reservoir hosts of human babesiosis on Nantucket Island. Am J Trop Med Hyg. 1981;30:560–5 . 7020449 10.4269/ajtmh.1981.30.560

[9] Krause PJ, Telford SR III, Ryan R, Hurta AB, Kwasnik I, Luger S, Geographical and temporal distribution of babesial infection in Connecticut. J Clin Microbiol. 1991;29:1–4 . 1993742 10.1128/jcm.29.1.1-4.1991 PMC269691

[10] Menis M, Anderson SA, Izurieta HS, Kumar S, Burwen DR, Gibbs J, Babesiosis among elderly Medicare beneficiaries, United States, 2006–2008. Emerg Infect Dis. 2012;18:128–31 . 10.3201/eid1801.110305 22257500 PMC3310092

[11] Diuk-Wasser M, Vourc’h G, Cislo P, Gatewood Hoen A, Melton F, Hamer S, Field and climate-based model for predicting the density of host-seeking nymphal *Ixodes scapularis*, an important vector of tick-borne disease agents in the eastern United States. Glob Ecol Biogeogr. 2010;19:504–14 http://onlinelibrary.wiley.com/doi/10.1111/j.1466-8238.2010.00526.x/abstract?systemMessage=Wiley+Online+Library+will+be+disrupted+on+7+December+from+10%3A00-15%3A00+GMT+%2805%3A00-10%3A00+EST%29+for+essential+maintenance&userIsAuthenticated=false&deniedAccessCustomisedMessage=.

[12] Pepin KM, Eisen RJ, Mead PS, Piesman J, Gatewood AH, Barbour AG, Geographic variation in the relationship between human Lyme disease incidence and density of infected host-seeking *Ixodes scapularis* nymphs in the eastern United States. Am J Trop Med Hyg. 2012;86:1062–71 . 10.4269/ajtmh.2012.11-0630 22665620 PMC3366524

[13] Mather TN, Nicholson MC, Donnelly EF, Matyas BT. Entomologic index for human risk of Lyme disease. Am J Epidemiol. 1996;144:1066–9 . 10.1093/oxfordjournals.aje.a008879 8942438

[14] Western KA, Benson GD, Gleason NN, Healy GR, Schultz MG. Babesiosis in a Massachusetts resident. N Engl J Med. 1970;283:854–6 . 10.1056/NEJM197010152831607 4989787

[15] Falco RC, McKenna DF, Daniels TJ, Nadelman RB, Nowakowski J, Fish D, Temporal relation between *Ixodes scapularis* abundance and risk for Lyme disease associated with erythema migrans. Am J Epidemiol. 1999;149:771–6 . 10.1093/oxfordjournals.aje.a009886 10206627

[16] Piesman J, Mather TN, Sinsky RJ, Spielman A. Duration of tick attachment and *Borrelia burgdorferi* transmission. J Clin Microbiol. 1987;25:557–8 . 3571459 10.1128/jcm.25.3.557-558.1987 PMC265989

[17] Falco RC, Fish D. A comparison of methods for sampling the deer tick, *Ixodes dammini*, in a Lyme-disease endemic area. Exp Appl Acarol. 1992;14:165–73 . 10.1007/BF01219108 1638929

[18] Durden LA, Keirans JE. Nymphs of the genus *Ixodes* (Acari:Ixodidae) of the United States: taxonomy, identification key, distribution, hosts and medical/veterinary importance. Lanham (MD): Entomological Society of America; 1996.

[19] Beati L, Keirans JE. Analysis of the systematic relationships among ticks of the genera *Rhipicephalus* and *Boophilus* (Acari: Ixodidae) based on mitochondrial 12S ribosomal DNA gene sequences and morphological characters. J Parasitol. 2001;87:32–48 . 11227901 10.1645/0022-3395(2001)087[0032:AOTSRA]2.0.CO;2

[20] Liveris D, Gazumyan A, Schwartz I. Molecular typing of *Borrelia burgdorferi* sensu lato by PCR-restriction fragment length polymorphism analysis. J Clin Microbiol. 1995;33:589–95 . 7751362 10.1128/jcm.33.3.589-595.1995 PMC227995

[21] Travinsky B, Bunikis J, Barbour AG. Geographic differences in genetic locus linkages for *Borrelia burgdorferi.* Emerg Infect Dis. 2010;16:1147–50 . 10.3201/eid1607.091452 20587192 PMC3321895

[22] Rollend L, Bent SJ, Krause PJ, Usmani-Brown S, Steeves TK, States SL, Quantitative PCR for detection of *Babesia microti* in *Ixodes scapularis* ticks and in human blood. Vector Borne Zoonotic Dis. 2013;13:784–90 . 10.1089/vbz.2011.0935 24107203 PMC3822370

[23] Connecticut Department of Public Health. Disease statistics. 2012 [cited 2012 May 2].http://www.ct.gov/dph/site/default.asp

[24] Massachusetts Department of Public Health. Surveillance data. 2012 [cited 2012 Jun]. http://www.mass.gov/eohhs/researcher/physical-health/diseases-and-conditions/communicable-diseases/public-health-cdc-babesiosis-surveillance.html

[25] Anderson JF, Mintz ED, Gadbaw JJ, Magnarelli LA. *Babesia microti*, human babesiosis, and *Borrelia burgdorferi* in Connecticut. J Clin Microbiol. 1991;29:2779–83 . 1757548 10.1128/jcm.29.12.2779-2783.1991 PMC270432

[26] Goethert HK, Lubelcyzk C, LaCombe E, Holman M, Rand P, Smith RP, Enzootic *Babesia microti* in Maine. J Parasitol. 2003;89:1069–71. 10.1645/GE-3149RN 14627162

[27] Spielman A. The emergence of Lyme disease and human babesiosis in a changing environment. Ann N Y Acad Sci. 1994;740:146–56. 10.1111/j.1749-6632.1994.tb19865.x 7840446

[28] Piesman J, Mather TN, Donahue JG, Levine J, Campbell JD, Karakashian SJ, Comparative prevalence of *Babesia microti* and *Borrelia burgdorferi* in four populations of *Ixodes dammini* in eastern Massachusetts. Acta Trop. 1986;43:263–70 . 2430433

[29] Varde S, Beckley J, Schwartz I. Prevalence of tick-borne pathogens in *Ixodes scapularis* in a rural New Jersey County. Emerg Infect Dis. 1998;4:97–9. 10.3201/eid0401.980113 9452402 PMC2627663

[30] Adelson ME, Rao R-VS, Tilton RC, Cabets K, Eskow E, Fein L, Prevalence of *Borrelia burgdorferi, Bartonella* spp., *Babesia microti*, and *Anaplasma phagocytophila* in *Ixodes scapularis* ticks collected in northern New Jersey. J Clin Microbiol. 2004;42:2799–801. 10.1128/JCM.42.6.2799-2801.2004 15184475 PMC427842

[31] Joseph JT, Roy SS, Shams N, Visintainer P, Nadelman RB, Hosur S, Babesiosis in Lower Hudson Valley, New York, USA. Emerg Infect Dis. 2011;17:843–7. 10.3201/eid1705.101334 21529393 PMC3321771

[32] Rodgers SE, Mather TN. Human *Babesia microti* incidence and *Ixodes scapularis* distribution, Rhode Island, 1998–2004. Emerg Infect Dis. 2007;13:633–5. 10.3201/eid1304.061035 17553286 PMC2726110

[33] Krause PJ, McKay K, Gadbaw J, Christianson D, Closter L, Lepore T, Increasing health burden of human babesiosis in endemic sites. Am J Trop Med Hyg. 2003;68:431–6 . 12875292

[34] Iacopino V, Earnhart T. Life-threatening babesiosis in a woman from Wisconsin. Arch Intern Med. 1990;150:1527–8. 10.1001/archinte.1990.00390190159027 2369251

[35] White DJ, Chang HG, Benach JL, Bosler EM, Meldrum SC, Means RG, The geographic spread and temporal increase of the Lyme-disease epidemic. JAMA. 1991;266:1230–6. 10.1001/jama.1991.03470090064033 1870248

[36] Mather TN, Telford SR. III Sr, Moore SI, Spielman A. *Borrelia burgdorferi* and *Babesia microti*: Efficiency of transmission from reservoirs to vector ticks (*Ixodes dammini*). Exp Parasitol. 1990;70:55–61. 10.1016/0014-4894(90)90085-Q 2295326

[37] Piesman J, Mather TN, Dammin GJ, Telford SR III, Lastavica CC, Spielman A. Seasonal variation of transmission risk of Lyme-disease and human babesiosis. Am J Epidemiol. 1987;126:1187–9 . 3687924 10.1093/oxfordjournals.aje.a114757

[38] Mather TN, Nicholson MC, Hu RJ, Miller NJ. Entomological correlates of *Babesia microti* prevalence in an area where *Ixodes scapularis* (Acari: Ixodidae) is endemic. J Med Entomol. 1996;33:866–70 . 8840700 10.1093/jmedent/33.5.866

[39] Piesman J, Spielman A. Human babesiosis on Nantucket Island: prevalence of *Babesia microti* in ticks. Am J Trop Med Hyg. 1980;29:742–6 . 7435782 10.4269/ajtmh.1980.29.742

[40] Steere AC, Sikand VK, Schoen RT, Nowakowski J. Asymptomatic infection with *Borrelia burgdorferi.* Clin Infect Dis. 2003;37:528–32 . 10.1086/376914 12905137

